# Supplementary material for: Evaluation of Prebiotic Potential of Crude Polysaccharides Extracted from Wild Lentinus polychrous and Lentinus squarrosulus and Their Application for a Formulation of a Novel Lyophilized Synbiotic
Source: Foods. 2024 Jan 16;13(2):287. doi: 10.3390/foods13020287 (PMC10815080; doi:10.3390/foods13020287)
Supplement: Supplementary file 1 [file foods-13-00287-s001.zip › Supplement data for Figure 5 (Table S2 to Table S4).pdf]

Supplement data for Figure 5 (Table S2 to Table S4)

Table S2 Contents of reducing sugar releasing in gastric buffer pH 1 and 5 (Raw data)

Polysaccharides	Polysaccharides (ug/mg)	Initial reducing sugar (ug/mg)			Reducing sugar (ug/mg) in pH 1			Reducing sugar (ug/mg) in pH 5		
		rep1	rep2	rep3	rep1	rep2	rep3	rep1	rep2	rep3
**Inulin**	836.37±19.58	10.56	12.45	8.53	56.81	58.56	63.45	50.71	55.56	53.05
**CPS_UBU_LS1**	368.40±31.32	35.89	30.48	32.15	65.75	60.5	62.54	62.75	58.03	60.4
**CPS_UBU_LP2**	418.46±15.21	25.15	22.46	26.5	50.9	48.6	52.47	53.7	50.16	50.52

Table S3 The percent of polysaccharide hydrolysis in gastric buffer pH 1 and 5 (Raw data)

Polysaccharides	% of hydrolysis in pH 1			% of hydrolysis in pH 5		
	rep1	rep2	rep3	rep1	rep2	rep3
**Inulin**	5.54	5.75	6.33	4.81	5.39	5.09
**CPS_UBU_LS1**	8.93	7.51	8.06	8.12	6.84	7.48
**CPS_UBU_LP2**	6.26	4.28	5.33	6.93	4.70	4.80

Table S4 The percent of polysaccharide hydrolysis tolerance in gastric buffer pH 1 and 5 (Raw data)

Polysaccharides	% of hydrolysis tolerance in pH 1			% of hydrolysis tolerance in pH 5		
	rep1	rep2	rep3	rep1	rep2	rep3
**Inulin**	94.46	94.25	93.67	95.19	94.61	94.91
**CPS_UBU_LS1**	91.07	92.49	91.94	91.88	93.16	92.52
**CPS_UBU_LP2**	93.74	95.72	94.67	93.07	95.30	95.20
